# A protein interaction map for cell-cell adhesion regulators identifies DUSP23 as a novel phosphatase for β-catenin

**DOI:** 10.1038/srep27114

**Published:** 2016-06-03

**Authors:** Lisa Leon Gallegos, Mei Rosa Ng, Mathew E. Sowa, Laura M. Selfors, Anne White, Ioannis K. Zervantonakis, Pragya Singh, Sabin Dhakal, J. Wade Harper, Joan S. Brugge

**Affiliations:** 1 Cell Biology, Harvard Med School, Boston, MA, USA

## Abstract

Cell-cell adhesion is central to morphogenesis and maintenance of epithelial cell state. We previously identified 27 candidate cell-cell adhesion regulatory proteins (CCARPs) whose down-regulation disrupts epithelial cell-cell adhesion during collective migration. Using a protein interaction mapping strategy, we found that 18 CCARPs link to core components of adherens junctions or desmosomes. We further mapped linkages between the CCARPs and other known cell-cell adhesion proteins, including hits from recent screens uncovering novel components of E-cadherin adhesions. Mechanistic studies of one novel CCARP which links to multiple cell-cell adhesion proteins, the phosphatase DUSP23, revealed that it promotes dephosphorylation of β-catenin at Tyr 142 and enhances the interaction between α- and β-catenin. DUSP23 knockdown specifically diminished adhesion to E-cadherin without altering adhesion to fibronectin matrix proteins. Furthermore, DUSP23 knockdown produced “zipper-like” cell-cell adhesions, caused defects in transmission of polarization cues, and reduced coordination during collective migration. Thus, this study identifies multiple novel connections between proteins that regulate cell-cell interactions and provides evidence for a previously unrecognized role for DUSP23 in regulating E-cadherin adherens junctions through promoting the dephosphorylation of β-catenin.

Cell-cell adhesions are essential for developmental morphogenesis and maintenance of the epithelial cell state, and are mediated through specialized structures including adherens junctions, desmosomes and tight junctions. While adherens junctions (AJs) are nucleated by homotypic engagement of transmembrane E-cadherin molecules on adjacent cells, it is clear that E-cadherin molecules make up only a fraction of the total protein composition at these sites^1^,^2^. Therefore, the AJ has the molecular diversity to integrate the physical and chemical signals that regulate proliferation, survival, movement, and other behaviors of epithelial cells^3^,^4^,^5^. Similarly, transmembrane desmosomal cadherins initiate the formation of desmosomes and link these structures to other intracellular proteins. The dense packing of desmosomal cadherins has led to speculation that the role of these adhesions is primarily for mechanical support^1^; however, recent studies have also revealed more precise roles of desmosomes in regulating cell proliferation and differentiation^6^,^7^,^8^. As such, both adherens and desmosomal junctions are sites of intercellular connections that coordinate the actions of numerous proteins to promote the homeostasis of epithelial tissues.

Intracellularly, E-cadherin associates with the actin cytoskeleton dynamically through a number of known protein-protein interactions^1^. Association with the actin cytoskeleton and the correct positioning of the AJ requires binding of the cytoplasmic tail of E-cadherin to α- and β-catenin, and is refined by acto-myosin contractility, microtubule reorientation, phosphorylation events, the activity of Rho GTPases, and other factors^5^. Desmosomal cadherins associate with intermediate filaments via desmosomal catenins and desmoplakin^9^. Although much is known about the core adherens and desmosomal junction complexes, the full diversity of mechanisms involved in promoting and regulating cell-cell adhesion in epithelial cells has not been defined.

Recent siRNA screens^10^ and E-cadherin proximity methods^11^,^12^ have identified a number of new cell-cell adhesion proteins; however, elucidating how these proteins functionally regulate cell-cell adhesion would be greatly assisted by efforts to map their interconnectivity. We previously identified a subset of 27 genes whose perturbation disrupts cell-cell adhesion during collective migration^13^; many of these genes had no prior association with this process. In this study, we utilized a proteomics approach to identify high-confidence interacting proteins for the candidate cell-cell adhesion regulating proteins (CCARPs) encoded by these genes. The resulting interacting proteins serve as a ‘road-map’ that extensively links CCARPs to known cell-cell adhesion proteins and to each other. We elucidated a mechanism whereby one CCARP with multiple adhesion-relevant network connections, the phosphatase DUSP23, regulates cell-cell adhesion through tuning the interaction between α- and β-catenin. In addition, this study provides a rich connectivity map amongst both known and novel cell-cell adhesion regulatory proteins.

## Results and Discussion

### Mapping protein-protein interactions for cell-cell adhesion regulators

**Figure 1:**
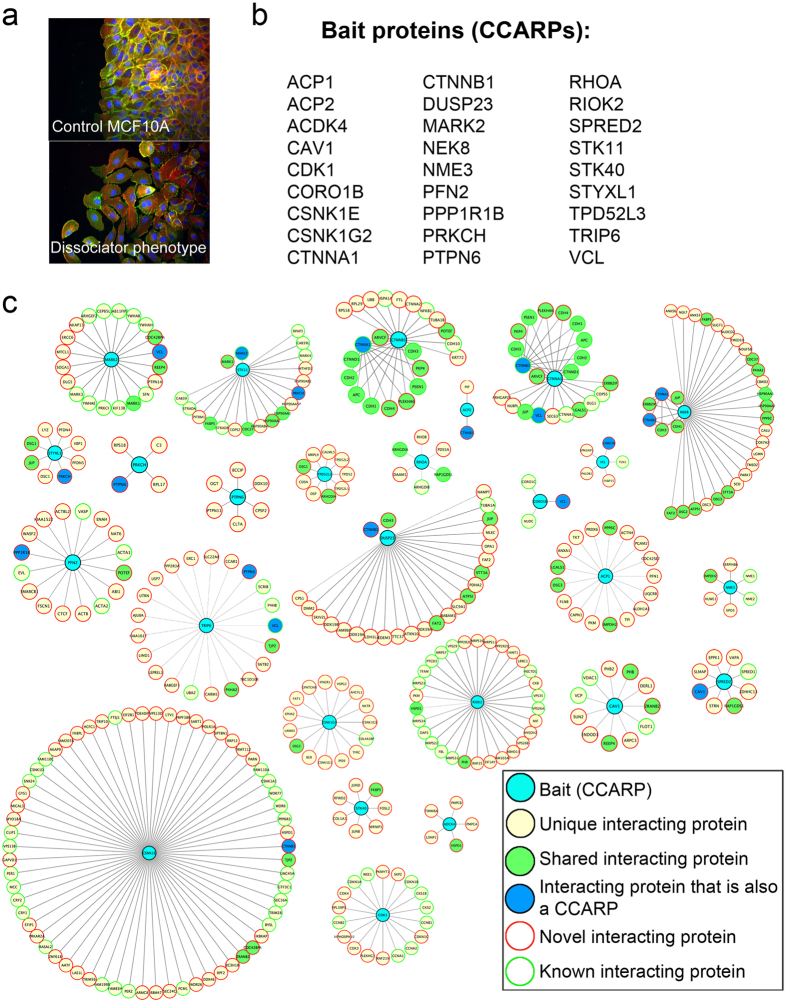
Protein interaction networks for Cell-Cell Adhesion Promoting Proteins (CCARPS). **(a)** Representative images of MCF10A cells migrating from right to left to fill a scratch wound. Cells were transfected with SMARTpool control siRNAs (top image) or siRNAs to ACP2, the latter shown to represent the dissociator phenotype (bottom image). **(b)** List of genes whose siRNA knockdown causes a dissociator phenotype^13^. **(c)** Interacting proteins for each of the proteins encoded by the genes listed in B; Bait proteins are shaded in cyan. Unique interacting proteins for each bait are shaded in pale yellow, interacting proteins common to two or more baits are shaded in green, and interacting proteins that were among the original baits are shaded in blue. Novel interacting proteins are outlined in red and previously identified interacting proteins are outlined in green.

A previous large-scale study of genes that regulate collective cell migration identified 27 genes which, when knocked down by siRNA, resulted in dissociation of cells at the leading edge of a collectively migrating MCF10A monolayer (Fig. 1a,b; Supplemental Fig. S1)^13^. Although known cell-cell adhesion proteins such as α- and β-catenin are amongst these hits, many of these genes had never before been associated with cell-cell adhesion. In order to generate a framework to investigate the mechanisms by which the candidate cell-cell adhesion-regulating proteins (CCARPs) encoded by these 27 genes impact cell-cell adhesion, we undertook a protein interaction mapping strategy that has been previously used to identify functional linkages en masse for cellular processes such as deubiquitination and autophagy^14^,^15^. For this strategy, we constructed retroviral expression vectors encoding FLAG-HA-tagged variants of each CCARP and transduced these into MCF10A cells to generate stable cell lines. We then carried out a series of immunoprecipitation experiments to pull down the different tagged CCARPs (‘bait’ proteins) with their associated interacting proteins to generate a set of parallel LC-MS/MS datasets. The identified peptides for each immunoprecipitate (IP) were analyzed using a modified version of the Comparative Proteomics Analysis Software Suite (CompPASS) to generate a series of specificity metrics for each interacting protein [including the normalized weighted D (WD^N^) score, which incorporates the frequency, abundance and reproducibility of each interaction] to assist with identification of significant interactions for each of the 27 CCARP baits^15^. In this method, 64 parallel IP-MS experiments involving unrelated bait proteins were used to build a “stats” table for CompPASS analysis. Each IP-MS experiment serves as a control for all others.

Proteins detected in the CCARP IPs with WD^N^ scores ≥ 1 were automatically considered “high confidence interacting proteins” (HCIPs, 304 hits across the 27 baits). However, we noticed that many previously reported interactions for known cell-cell adhesion proteins fell just below the very stringent top WD^N^ score cutoff of 1, which includes only the top 2% of WD^N^ scores. So, we also included proteins with WD^N^ scores ≥ 0.41 (top 5%) or Z-score ≥ 3.48 (top 5%) as HCIPs, if they either were previously associated with cell-cell adhesion, or were reported to interact with the bait in a prior study (see Methods for more information). In total, we identified 284 novel and 116 previously identified protein interactions. The HCIPs identified for each of the CCARPs are shown in Fig. 1c and listed in Supplemental Table S1. Supplemental Table S1 additionally details which interactions had a WD^N^ score ≥ 1, and which were included by the limited top 5% cutoffs described above. Supplemental Table S4 contains all the scored interactors for each IP; as a control, no interacting proteins in the IP of HA-GFP had WD^N^ scores ≥ 0.41 or Z-scores ≥ 3.48. As with all co-immunoprecipitation experiments, this method identifies complexes of proteins that interact stably through either direct or indirect protein contacts.

Multiple known AJ protein interactions were detected within our dataset of HCIPs. For example, we identified reciprocal interactions between α- and β-catenin, and linkages to of these proteins to δ-catenin, E-cadherin, and other cadherins. One noteworthy HCIP detected for α-catenin is the context-dependent interacting protein, vinculin. Because vinculin only interacts with the AJ complex at cell-cell junctions under tension^16^,^17^, the detection of this interaction above the WD^N^ scoring cutoff also provides evidence that our dataset contains complexes consisting of bona fide junctional proteins. We additionally detected several other known interactors for α- and β-catenin, such as presenillin and APC. Other known adhesion/polarity/cytoskeletal regulatory complexes were also detected, including the MARK2 (PAR1)-STK11 (LKB1) kinase-substrate complex^18^, RhoA-RhoGDI^19^, profilin2-VASP/EVL-actin^20^, and vinculin-talin^21^ interactions. The detection of known cell-cell junction complexes and cytoskeletal regulatory complexes amongst the CCARPs supports the validity of this approach.

In addition, our HCIPs linked many CCARPs together in protein complexes. For example, vinculin and CORO1B were reciprocally detected as HCIPs for each other. CORO1B had previously been identified in E-cadherin junction complexes^11^, however, it had not been linked to these adhesion complexes through vinculin. Vinculin also interacted with the CCARP polarity protein MARK2 (PAR1); although the literature strongly supports their roles in promoting cell-cell adhesion^22^,^23^, their interaction has not been reported. NEK8 was found to interact with α-catenin and β-catenin, as well as E- and P-cadherin, suggesting that this protein, which is mutated in polycystic kidney disease^24^ and regulates organ growth through the Hippo pathway^25^, interacts with AJs. Additional interactions amongst the CCARPs included PTPN6 (SHP1)-PRKCH (PKCη), STYXL1- PRKCH (PKCη), CAV1-SPRED2, PFN2-PPP1R1B, vinculin-TRIP6, vinculin-PTPN6 (SHP1), and others; CCARPs that are HCIPs for another bait are denoted by dark blue symbols in Fig. 1c. The identification of discrete novel protein complexes amongst the CCARPs further suggests which of these proteins may cooperate together to regulate cell-cell adhesion.

**Figure 2:**
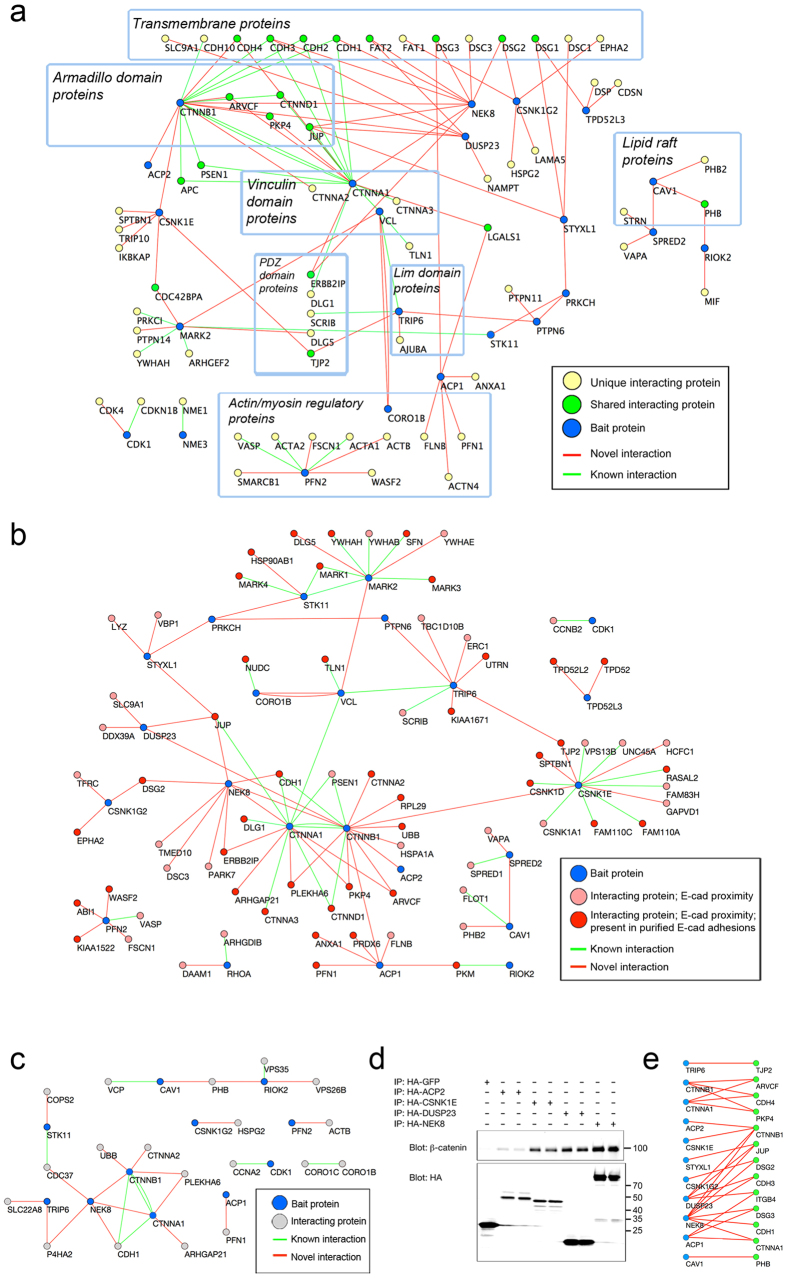
Interactions linking CCARPs to known adhesion proteins. **(a)** Selected network proteins that are annotated for cell-cell adhesion gene ontology and their associated bait proteins. Baits are shaded in blue, unique interacting proteins are shaded in pale yellow, and interacting proteins common to two or more baits are shaded in green. Edges connecting proteins represent interactions between two proteins; novel interactions are in red, and known interactions are in green. Nodes are organized according to functional pathways and/or domain structures. **(b)** Selected network proteins that were found in proximity to E-cadherin using biotin ligase screening methods or were found in E-cadherin adhesion plaques^11^. Baits are shaded in blue, interacting proteins that were found in proximity to E-cadherin are shaded in rose, and interacting proteins that were both found in proximity to E-cadherin and were also present in purified E-cadherin adhesion plaques are shaded in red. Edges connecting proteins represent interactions between two proteins; novel interactions are in red, and known interactions are in green. **(c)** Selected network proteins that were found in a functional screen for adhesion regulators in *Drosophila* S2 cells^10^. Baits are shaded in blue, interacting proteins are shaded in grey. Edges connecting proteins represent interactions between two proteins; novel interactions are in red, and known interactions are in green. **(d)** Representative blot for validation of novel protein interactions by IP/MS. HA-tagged proteins were immunoprecipitated from MCF10A cells, and analyzed for associated β-catenin (top blot) and for successful IP of the tagged bait protein (bottom blot). **(e)** Schematic diagram of validated novel interactions between bait proteins (blue nodes) and interacting proteins identified by IP/MS (green nodes).

We next systematically assessed the extent to which CCARPs connected to other proteins that were previously functionally associated with cell-cell adhesion (Supplemental Table S2 lists cell-cell adhesion gene ontology terms). We found that 98 of the proteins that co-precipitated with the CCARPs are previously annotated cell-cell adhesion proteins (Fig. 2a); of these interactions, 28 were known (green connecting lines) and 70 had not previously been described (red connecting lines). Notably, 12 of the 27 CCARPs either co-precipitated with components of the classical cadherin adhesion complex or were connected through one intermediate interacting protein, and six of the CCARPs could similarly be connected to desmosomal and/or atypical cadherin adhesion complexes (Fig. 2a). For example, linkages between three Tyr phosphatases – ACP1, ACP2, and DUSP23 – and the AJ complex were identified, only one of which, ACP1, was previously associated with this complex^26^,^27^. These associations of CCARPs with known adhesion proteins, together with the CCARP-CCARP complexes we also identified, significantly expands the number of candidate regulators of cell-cell adhesion and will assist mechanistic studies of cell-cell adhesion.

In addition to identifying interactions between proteins that regulate cell-cell adhesion functionally, we further sought to classify interactions that are likely to take place at E-cadherin cell-cell junctions. For this purpose, we resourced a recent report which identified proteins by mass spectrometry that were either 1) found in proximity to E-cadherin in cells using a BirA biotin ligase method or 2) isolated from adhesion plaques that were formed by cells adhering to glass coated with E-cadherin^11^. Amongst the E-cadherin adhesion-associated proteins identified in this prior screen (Fig. 2b), we identified 39 interactions that had previously been reported in the literature and an additional 64 novel interactions. Most notable are the interactors represented by red nodes; these proteins were both present at E-cadherin adhesion plaques and found in proximity to E-cadherin. Thus, interactions with these proteins have the strongest likelihood of occurring at E-cadherin adhesions. Additional interactions were mapped for 20 of the hits identified in a recent functional screen for cell-cell adhesion regulators in *Drosophila* S2 cells^10^ (Fig. 2c). These interactions may point toward evolutionarily conserved adhesion mechanisms. Interestingly, we identified interactions linking two novel cell-cell adhesion proteins identified in both screens, PLEKHA6 and ARHGAP21, to the core α-catenin/β-catenin adhesion complex. ARHGAP21 was recently shown to transiently translocate to the membrane shortly after initiation of cell-cell adhesion^28^, but its linkage to core cell-cell adhesion proteins was not previously identified. Supplemental Table S3 contains a list of all the interactions for cell-cell adhesion proteins identified in previous functional screens^10^ and in E-cadherin proximity assays^11^,^12^.

After referencing our dataset to previously reported protein-protein interactions and experimentally validated cell-cell adhesion proteins, we further selected 27 novel interactions with known cell-cell adhesion proteins to validate by IP/western blot. These interactions were selected based on 1) identification of a suitable antibody to detect the HCIP and 2) the presence of connections between the HCIP and multiple baits, to maximize our validation efforts. We validated 25 out of 27 of these novel interactions with known cell-cell adhesion proteins by immunoprecipitation and western blot (92.5%, Fig. 2d,e; Supplemental Fig. S2), showing that the bait protein, and not HA-tagged GFP, pulled down the interacting protein. Three of the interactions that were validated by western blot (ACP1-CTNNA1, DUSP23-ITGB4, and ACP1-ITGB4) fell just beneath the top 5% scoring cutoffs for proteins with cell-cell adhesion gene ontology. This supports the stringency of our cutoffs and suggests that additional relevant protein interactions may fall beneath the WD^N^ and Z-score cutoffs. The full set of scored interactors for each of the baits can be found in Supplemental Table S4.

Although we have made efforts to identify the most adhesion-relevant interactions amongst our dataset, many protein interactions we identified clearly take place at other regions of the cell. For example, one CCARP, ADCK4, interacted exclusively with mitochondrial proteins. While these interactions indicate that ADCK4 may be irrelevant to cell-cell adhesion, the loss-of-adhesion phenotype induced by all four individual siRNAs targeting ADCK4^13^ suggests an alternate hypothesis that CCARPs such as ADCK4 may be potential regulators of signaling cross-talk between cell-cell adhesions and other cellular structures. A recent example of this crosstalk is a report of the atypical cadherin FAT participating in the regulation of mitochondrial metabolism^29^. Taken together, these efforts to map interactions between known and newly described cell-cell adhesion proteins will guide future studies to understand how cell-cell junctions are regulated, and may represent a starting point for new discoveries in how cell-cell adhesions interface with multiple aspects of cell biology.

### DUSP23 controls the association between α- and β-catenin by promoting dephosphorylation of Tyr142

**Figure 3:**
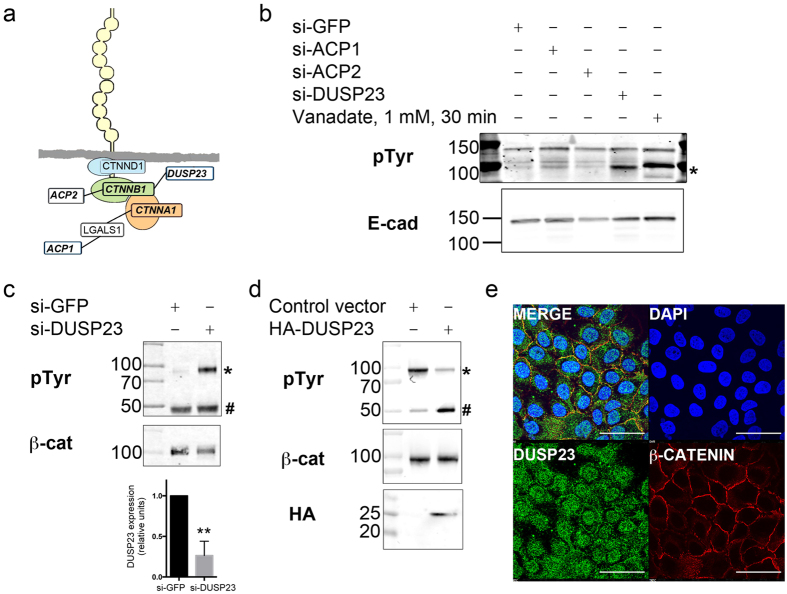
DUSP23 regulates Tyr phosphorylation of β-catenin and co-localizes with it at cell-cell junctions. **(a)** Diagram of connections between the cadherin-catenin complex and three Tyr phosphatases. **(b)** Analysis of changes in Tyr phosphorylation of E-cadherin-associated proteins. E-cadherin was immunoprecipitated from cells transfected with siRNA to GFP (negative control), ACP1, ACP2, and DUSP23, or from cells treated with Vanadate (positive control). Blots were probed with anti-E-cadherin (bottom) or anti-pTyr antibody (top). *Denotes pTyr band in the DUSP23 knockdown lane at 100 kDa. **(c)** Immunoblot analysis of pTyr β-catenin immunoprecipitated from MCF10A cells treated with siRNA to GFP or siRNA smartpool to DUSP23; blots were probed with anti-pTyr (top) or anti-β-catenin (bottom) antibody. DUSP23 knockdown was monitored by QPCR (bottom graph). *Denotes pTyr signal and ^#^denotes cross-reactive IgG protein. Immunoblots are representative of 3 experiments. Graph is a quantification of 2 independent experiments carried out in triplicate. Error bar indicates S. D. **p < 0.03. **(d)** Immunoblot analysis of pTyr of β-catenin immunoprecipitated from MCF10A cells expressing control plasmid or HA-tagged DUSP23; blots were probed with anti-pTyr (top), anti-β-catenin (middle), or anti-HA (bottom) antibody. *Denotes pTyr signal and ^#^denotes cross-reactive IgG protein. Immunoblots are representative of 3 experiments**. (e)** Immunofluorescence staining of MCF10A cells using rabbit antibody to DUSP23 (green, lower left) and mouse antibody to β-catenin (CTNNB1, red, lower right). DAPI (upper right) stains the nuclei, and merged image (top left) shows co-localization of DUSP23 with β-catenin. Scale bar represents 50 µm.

We next sought to explore the novel linkages between under-characterized CCARPs and the core E-cadherin adhesion complex to discover mechanisms by which these proteins promote cell-cell adhesion. Phosphorylation of the AJ complex by Src family kinases has been well described^30^,^31^, and this is known to influence the connectivity amongst the proteins within the AJ complex and the functional stability of the AJ^32^,^33^. Hence, we focused our efforts on the three CCARP phosphatases – ACP1, ACP2, and DUSP23 – that linked to the AJ complex (Fig. 3a). To examine whether any of the three phosphatases regulate the tyrosine phosphorylation status of E-cadherin-associated proteins, we immunoprecipitated E-cadherin from cells in which each of the phosphatases had been individually knocked down by siRNA and blotted for E-cadherin-associated tyrosine phosphorylated proteins (Fig. 3b). Treatment with the tyrosine phosphatase inhibitor vanadate was included as a positive control. Consistent with previous studies implicating ACP1 (or LMW-PTP) in dephosphorylation of β-catenin^26^, we saw a slight increase in phosphotyrosine (pTyr) signal for a band migrating near 100 kDa, which corresponds to the size of α-, β-, and δ-catenin, in cells with ACP1 knockdown. However, a greater increase in pTyr at 100 kDa was observed in DUSP23 knockdown cells.

DUSP23/VHZ is the smallest catalytically active dual-specificity phosphatase^34^. While DUSP23 has been reported to dephosphorylate peptides containing either Thr or Tyr residues *in vitro*^35^, the substrate/product binding properties identified in the crystal structure of DUSP23 bound to substrate peptide are similar to those of the Tyr phosphatase PTP1b^34^. In addition to our previous screen which revealed a loss of cell-cell adhesion in MCF10A cells upon knockdown of DUSP23, a functional screen for cell-cell adhesion regulators in *Drosophila* S2 cells identified a candidate ortholog of DUSP23, cdc14^10^, as a gene that is required for cell-cell adhesion. Thus, DUSP23 is a catalytically active phosphatase that is capable of dephosphorylating Tyr residues and has been implicated in two screens as a potential cell-cell adhesion regulator. However, the mechanism by which DUSP23 regulates cell-cell adhesion is unknown.

Given that the candidate100-kDa E-cadherin-associated potential DUSP23 substrate is similar in size to α-, β-, and δ-catenin, we next examined whether DUSP23 regulates tyrosine phosphorylation of these core components of the E-cadherin adhesion machinery. Knockdown of DUSP23 caused a strong increase in the tyrosine phosphorylation of β-catenin (Fig. 3c), but had no effect on phosphorylation of α- or δ- catenin (Supplemental Fig. S3a). Conversely, cells expressing HA-DUSP23 showed reduced levels of pTyr β-catenin (Fig. 3d). We also generated a polyclonal antibody to DUSP23 and found that a fraction of endogenous DUSP23 co-localizes with β-catenin at cell-cell adhesions (Fig. 3e). Thus, DUSP23 co-localizes with and regulates the tyrosine phosphorylation of β-catenin.

**Figure 4:**
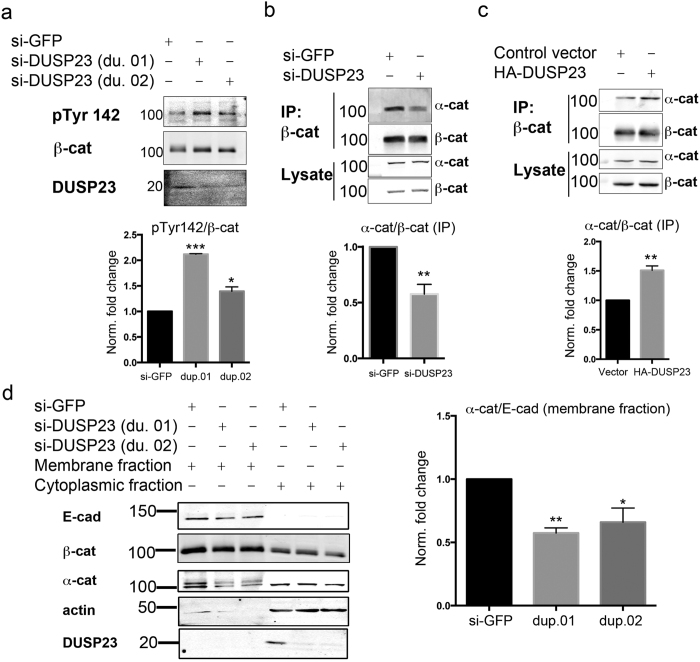
DUSP23 regulates the association of α- and β-catenin at cell membranes through Tyr 142 of β-catenin. **(a)** Immunoblot analysis of pTyr 142 β-catenin immunoprecipitated from MCF10A cells transfected with siRNA to GFP (control) or DUSP23 (two duplexes; du. 01 and du. 02); blots were probed with anti-pTyr-142 β-catenin (top), anti-β-catenin (middle), and anti-DUSP23 (bottom) antibody. Graph is a quantification of two independent experiments; ***p < 0.001, *p < 0.04. **(b)** Immunoblot analysis of α-catenin associated with β-catenin from MCF10A cells transfected with siRNA to GFP (control) or siRNA smartpool to DUSP23. Top blots are of β-catenin immunoprecipitation complexes; bottom blots are of lysates. Graph is a quantification of three independent experiments; **p < 0.008. **(c)** Immunoblot analysis of α-catenin associated with β-catenin from MCF10A cells expressing control plasmid or HA-tagged DUSP23. Top blots are of β-catenin immunoprecipitation complexes; bottom blots are of lysates. Graph is a quantification of three independent experiments; **p < 0.003. **(d)** Immunoblot analysis of membrane and cytoplasmic fractions of cells transfected with siRNA to GFP (control) or DUSP23 (two duplexes; du. 01 and du. 02). Blots were probed with anti-E-Cadherin antibody as a marker for membrane-associated protein complexes, and with actin as a marker for cytoplasmic protein complexes. Quantification of α-catenin protein levels in the membrane fraction (normalized to E-cadherin levels) across three independent experiments is shown at right. **p < 0.0001, *p < 0.02. All error bars indicate SEM.

Multiple Tyr phosphorylation sites on β-catenin have been mapped and are known to distinctly impact β-catenin association with interacting proteins^33^. Using site-specific antibodies to previously described Tyr phosphorylation sites on β-catenin, we carried out knockdown studies to determine which site(s) were regulated by DUSP23. DUSP23 knockdown did not affect phosphorylation of Tyr 654, a site known to regulate the association between β-catenin and E-cadherin^30^ (Supplemental Fig. S3b). Since phosphorylation of Tyr 654 would inhibit the binding of β-catenin to E-cadherin, our observation that DUSP23 knockdown promotes phosphorylation of an *E-cadherin-associated* protein (Fig. 3b) is consistent with DUSP23 regulating a phosphorylation site other than Tyr 654. Indeed, DUSP23 knockdown caused an increase in the levels of pTyr 142, a site known to regulate the interaction between α- and β-catenin (Fig. 4a).

Phosphorylation of Tyr 142 by Fer/Fyn kinases has been shown to disrupt the interaction between α- and β-catenin and lead to weakening of cell-cell junctions^31^. Consistent with the ability of DUSP23 to regulate pTyr 142, knockdown of DUSP23 decreased the amount of α-catenin that co-immunoprecipitated with β-catenin (Fig. 4b), and overexpression of DUSP23 led to an enhancement of α- and β-catenin association (Fig. 4c). Interestingly, pTyr 86 levels also increased with DUSP23 knockdown (Supplemental Fig. S3a). This site is only accessible to protein kinases when α-catenin is dissociated from β-catenin^36^; thus, the evidence that knockdown of DUSP23 increased pTyr 86 provides additional support that DUSP23 knockdown decreases the binding between α- and β-catenin.

To further examine whether DUSP23 affects the association of α-catenin with the membrane-localized E-cadherin/catenin complex, we assessed the effects of DUSP23 knockdown on the presence of α-catenin in membrane-enriched and cytoplasmic fractions of MCF10A lysates. Knockdown of DUSP23 decreased the amount of α-catenin present in the membrane fraction (Fig. 4d), without significantly changing the amount of β-catenin present in this fraction (Fig. S4). Thus, our results support a model whereby DUSP23 knockdown increases the phosphorylation of Tyr 142 of β-catenin, leading to a decreased association between α-catenin and the membrane-localized E-cadherin/β-catenin adhesion complex. Given the known role of phosphorylation of Tyr 142 in weakening the interaction of β-catenin and α-catenin, the enhanced phosphorylation of Tyr 142 and Tyr 86 after DUSP23 knockdown, and the ability of DUSP23 alterations to affect the association of α-catenin to both β-catenin and the membrane fraction of cells, our results support the conclusion that DUSP23 specifically regulates cell-cell adhesion through promoting the dephosphorylation of Tyr 142.

### DUSP23 knockdown specifically reduces E-cadherin adhesion

**Figure 5:**
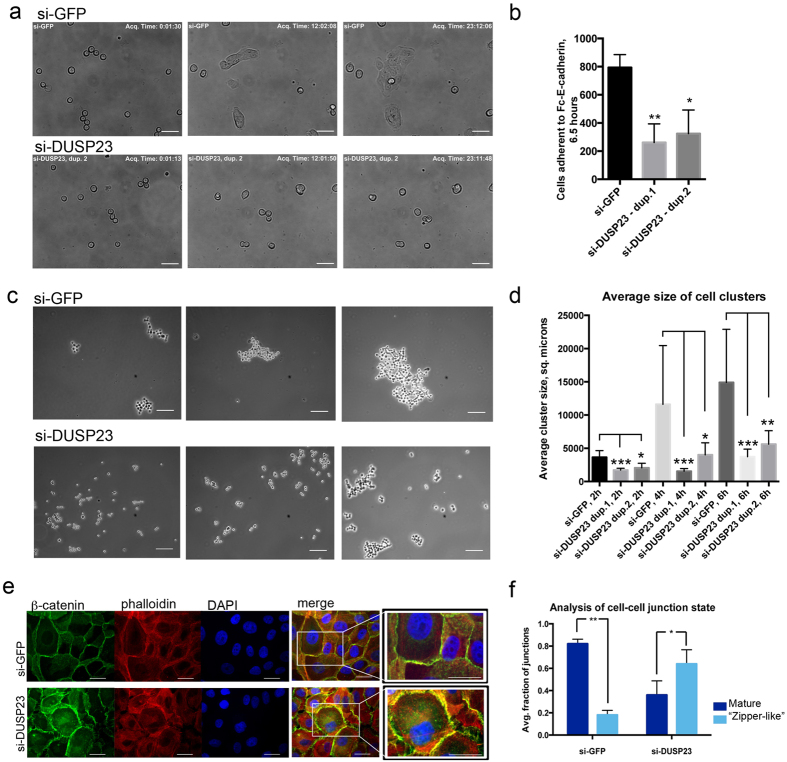
DUSP23 specifically regulates cell-cell adhesion and not matrix adhesion. **(a)** Images of MCF10A cells spreading on Fc-E-cadherin from Supplemental movies S1, S2, S3. Top images are of control cells (si-GFP) and bottom images are of cells transduced with duplex targeting DUSP23. From left to right, time points shown are 0, 12, and 24 hours after replating. Scale bar indicates 50 µm. **(b)** Adhesion assay of MCF10A cells to glass coated with Fc-E-cadherin. Graph shows quantification of control cells (si-GFP), or DUSP23 knockdown cells (si-DUSP23, two different duplexes) remaining adherent to E-cadherin coated surface. N = 3 wells across two independent experiments; error bars represent S. D. **p = 0.005, *p = 0.01 using multiple t-tests. **(c)** Representative images of MCF10A cell aggregates from “hanging drop” cell-cell adhesion assays. Top images are of control cells (si-GFP) and bottom images are of cells transduced with duplex targeting DUSP23. From left to right, time points shown are 2 h, 4 h, and 6 h. Scale bar indicates 100 µm. **(d)** Quantification of average sizes of cell aggregates shown in (**c**) for control MCF10A cells (si-GFP) or for DUSP23 knockdown cells (si-DUSP23, two different duplexes). N = 6 wells across two independent experiments; error bars represent 95% confidence interval for the mean. *p = 0.01, **p < 0.007, ***p < 0.0005 using multiple t-tests. **(e)** Images of confluent monolayers of MCF10A cells transfected with siRNA to GFP (control) or siRNA smartpool targeting DUSP23, and stained with antibody to β-catenin (green), phalloidin to visualize actin (red), and DAPI to visualize nuclei (blue). Scale bars represent 25 µm. Merged images are to the right, with an inset showing a larger magnification of cell-cell contact sites. **(f)** Line-scan analysis of cell-cell junctions of control and DUSP23 knockdown cells is presented in the graph. Over 50 cells were analyzed from 2 independent experiments; error bars represent S. D. **p = 0.003, *p = 0.04 using multiple t-tests.

Because alterations in cell-matrix adhesion strength could also contribute to a cell scattering phenotype during collective migration, we sought to further clarify whether the effects of DUSP23 knockdown were mediated specifically by alterations in E-cadherin-mediated adhesion. For this experiment, cells transduced with control siRNA, or two different siRNA duplexes targeting DUSP23, were plated on glass coated with purified Fc-E-cadherin. Timelapse analysis shows that control cells are capable of spreading on glass coated with Fc-E-cadherin, while DUSP23 knockdown cells attach and spread to a lesser extent (Fig. 5a, Supplemental movies S1, S2, 3). In addition, an adhesion assay quantifying the number of cells attached to E-cadherin coated surfaces after washout of the unbound cells shows a decrease in the number of cells attached to glass upon DUSP23 knockdown (Fig. 5b). In contrast, adhesion assays on fibronectin-coated glass showed similar attachment for control and DUSP23 knockdown cells (Fig. S5a). For both control and DUSP23 knockdown cells, the number of fibronectin-attached cells increased from 10–90 min, indicating that binding was not saturated at the time points used in these assays.

In addition to measuring adhesion to E-cadherin-coated surfaces, we carried out “hanging drop” assays in which suspensions of single MCF10A cells were placed in low-attachment round-bottom 96-well plates and allowed to form clusters over time; these conditions preclude the formation of cell-matrix adhesions, but allow the cells to form clusters through cell-cell adhesions. Figure 5c,d shows that cells with knockdown of DUSP23 formed clusters of significantly smaller size compared to cells transfected with control siRNA. The size of clusters increases over time, indicating that the conditions are appropriate for the detection of changes in cell-cell adhesion. Together, these data indicate that DUSP23 knockdown specifically induces a defect in E-cadherin-mediated cell-cell adhesion and does not alter cell-matrix interactions.

We further noted alterations in the appearance of cell-cell adhesions in monolayer cultures upon knockdown of DUSP23. While control cells formed mature junctions with β-catenin staining in uniform, linear patterns at the sites of cell-cell contact, DUSP23 knockdown cells formed less organized monolayers with a ‘zipper-like’ appearance reminiscent of the immature junctions formed in the early stages of cell-cell junction establishment^37^ (Fig. 5e). To quantify the extent of mature cell-cell contact formation, we carried out a line scan analysis of large stitched images of control or DUSP23 knockdown cells; a single peak of β-catenin staining intensity mid-way through the junction was assessed as a mature adhesion, while two peaks of staining intensity indicated a “zipper-like” adhesion (Supplemental Fig. S5b,c). Cells depleted of DUSP23 had a significant increase in the percentage of “zipper-like” cell-cell adhesions and a decrease in the percentage of mature cell-cell adhesions compared to the control cells (Fig. 5f). These data provide evidence that DUSP23 regulates cell-cell adhesion strength, and knockdown of DUSP23 causes the appearance of cells with “zipper-like” junctions.

### DUSP23 knockdown cells fail to coordinate movement and polarity cues during collective migration

**Figure 6:**
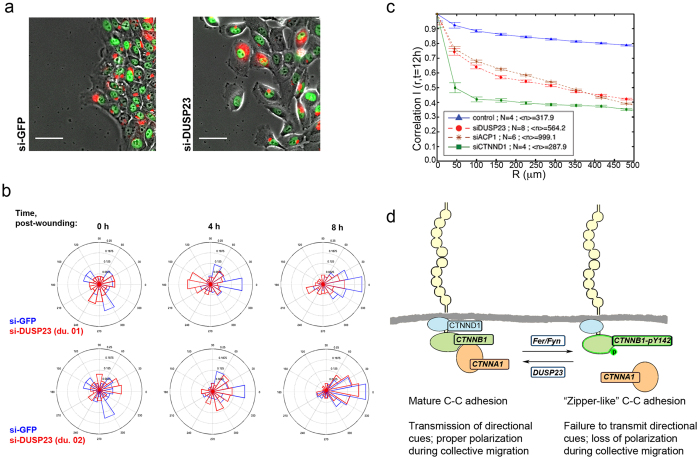
DUSP23 regulates polarization of cells toward the leading edge and coordination of cells during collective migration. **(a)** Assessment of polarization of cells toward the leading edge during collective cell migration at t = 0 h, 4 h, and 8 h after wounding. Images above show Golgi staining (red) relative to nuclei (green) at T = 4 h; scale bar represents 50 µm. **(b)** Rose plots to show quantification of Golgi orientation for si-GFP control cells (blue) and si-DUSP23 cells (red) for two independent siRNA duplexes (du. 01 and du. 02). Over 50 cells were analyzed from 2 independent experiments. **(c)** Assessment of the velocity correlation between MCF10A cells of a given radius during collective migration triggered by a scratch wound at t = 12 hours after wounding on glass. N = number of independent experiments for control (blue triangles), si-DUSP23 (red circles), si-ACP1 (brown asterisks) and si-CTNND1 (green squares) cells. Error bars indicate 95% confidence interval around the mean; non-overlapping error bars indicate results are statistically significantly different with α = 0.05. **(d)** Model for how DUSP23 impacts cell-cell adhesion by controlling pTyr 142 of β-catenin (green). E-cadherin is depicted in yellow, δ-catenin is in blue, and α-catenin is in orange.

A recent report from our laboratory showed that E-cadherin adhesions are sites of force transmission between cells that serve to coordinate their motion during collective migration^38^. To determine whether DUSP23 knockdown affects collective movements during epithelial cell migration, we first examined cell polarization toward the leading edge in a wound-healing assay. Knockdown of DUSP23 using two different siRNA duplexes either prevented (duplex 1) or delayed (duplex 2) polarization of MCF10A cells in the direction of the wound edge (Fig. 6a,b). The robustness of the effect of the individual siRNA duplexes in this assay correlates with their abilities to increase phosphorylation of Tyr 142 (Fig. 3f). We next assessed the velocity correlation, a measure of cell-cell coordination, as cells migrate to fill a wound. DUSP23 knockdown caused a reduction in velocity correlation compared to control cells (Fig. 6c, red circles vs. blue triangles). Interestingly, while DUSP23 depletion produced a phenotype that was less severe than knockdown of the core AJ complex protein δ-catenin (si-CTNND1, green squares), it was comparable to the phenotype caused by knockdown of another known phosphatase for β-catenin, LMW-PTP (si-ACP1, brown asterisks). Thus, these data suggest that DUSP23 is critical for maintaining adhesion at the leading edge, for coordinating movement within the monolayer, and for proper orientation of the cells in the direction of the wound edge (Fig. 6d).

We have previously shown that depletion of DUSP23 by siRNA promotes cell scattering during collective migration^13^. Here, we demonstrate that DUSP23 interacts with the canonical AJ complex, localizes at cell-cell junctions, and selectively regulates cell-cell and not cell-matrix adhesion. We further found that DUSP23 selectively regulates the phosphorylation of Tyr 142 of β-catenin, and the association between α- and β-catenin. While previous studies have demonstrated that pTyr 142 impacts α- and β-catenin association^31^,^36^ and β-catenin transcriptional activity^39^, the effects of uncoupling α- and β-catenin on epithelial cell-cell adhesion through this phosphorylation site have not been well-characterized. These studies support the conclusion that dephosphorylation of Tyr 142 of β-catenin at membranes regulates cell-cell adhesion strength. We also observed a reduction in cell polarization and cell-cell coordination during collective migration in DUSP23 knockdown cells. Previous studies established that reducing cell-cell adhesion through the knockdown of canonical cell-cell adhesion proteins is sufficient to produce these phenotypes^38^. Therefore, although it is possible that the loss of polarization and coordination could be mediated by targets of DUSP23 other than β-catenin, it is likely that these effects are due to weakened cell-cell adhesion through the mechanism we describe above. Our findings support a previously unrecognized role for the DUSP23 phosphatase in regulating β-catenin phosphorylation, adherens junction protein complexes, cell-cell adhesion strength, and coordination during collective cell migration.

### Summary

Although numerous elegant studies have identified and characterized many salient features of cell-cell adhesion, and recent studies have begun to uncover additional potential adhesion regulators^10^,^11^,^12^, identifying additional interactions between cell-cell adhesion proteins would greatly assist in understanding how these proteins regulate cell-cell junctions. In this study, we utilized a comparative IP/MS analysis^14^ to identify high-confidence protein interactions for each of the 27 proteins that we previously identified as candidate regulators of cell-cell adhesion. This led to the identification of linkages amongst the 27 proteins, as well as linkages to previously described cell-cell adhesion proteins. In follow-up studies, we identified DUSP23 as a novel phosphatase that regulates the connection between α- and β-catenin. Because cell-cell adhesions regulate so many aspects of cellular physiology that promote homeostatic behavior in tissues, it is not surprising that many tumors show loss of E-cadherin adhesions^40^,^41^, and that defects in desmosomal adhesion lead to diseases of skin and other epithelial tissues^9^. A broader characterization of proteins that are involved in cell-cell adhesion, bolstered by this study and other efforts to reveal the full spectrum of molecules involved in this process^10^,^11^,^12^, will contribute to our understanding of how cell-cell adhesion structures are regulated, and how their dysfunction contributes to disease states.

## Materials and Methods

### Plasmids and cell lines

Sequence-verified Gateway entry clones containing open reading frames for the dissociator genes were obtained from the human ORFeome collection or the Dana Farber/Harvard Cancer Center PlasmID Database, or were amplified by RT-PCR from MCF10A cells and cloned into pDONR223 (See Supplemental Table S5). Entry clones were recombined into the Gateway destination vector MSCV-N-Flag-HA-IRES-PURO (LTR-driven expression), with the exception of VCL, which was cloned into MSCV-C-Flag-HA-IRES-PURO, using LR clonase (Life Technologies) to generate expression plasmids. MCF10A cells (ATCC) were cultured as previously described (http://brugge.med.harvard.edu/ protocols). HEK293T cells (ATCC) were cultured in DMEM (Invitrogen), 10% fetal bovine serum (Gibco), 50 U/ml penicillin, and 50 μg/ml streptomycin.

### Virus production

Retroviruses produced by co-transfection of each expression plasmid with packaging vector pCL-Ampho into HEK293T cells as previously described^42^ were used to infect MCF10A cells for constitutive LTR-driven expression of the Flag-HA-protein.

### Antibodies

Mouse anti-β-catenin (CTNNB1, BD transduction laboratories, #610154), rabbit anti-α-catenin (CTNNA1, Sigma, #C2081), mouse anti-E-cadherin (CDH1, BD transduction laboratories, #610182), mouse anti-phospho-Tyr 4G10 (Millipore, #05-1050), rabbit anti-β-catenin phospho-Tyr142 (Abcam, #ab27798), anti-GM130 (BD transduction laboratories, #610823), rabbit anti-ARVCF (Bethyl Laboratories, #A303-310A), rabbit anti-CDH4 (Sigma, #HPA015613), rabbit anti-ERBB2IP (Sigma, #HPA048606), rabbit anti-PKP4 (Abcam, #ab128638), rabbit anti-PLEKHA6 (Sigma, #HPA028152), rabbit anti-PPP6C (Abcam, #ab70634), rabbit anti-PPP6R3 (Abcam, #ab72034), rabbit anti-HA (Cell signaling technologies, #3724), rabbit anti-TJP2 (ZO-2; Cell signaling technologies, #2847), mouse anti-JUP (Invitrogen, #12-8500), mouse anti-DSG2 (Sigma, #SAB4200466), mouse anti-p-cadherin (CDH3, BD biosciences, #610227), rabbit anti-DSG3(Abcam, #ab128927), mouse anti-PHB (NeoMarkers, #MS-261-PO), Rabbit anti-ITGB4 (Cell signaling technologies, #4707), mouse anti-Integrin β1 (BD biosciences, #552828). A polyclonal antibody against the peptide “NH2-RRLRPGSIETYEQEK-Cys” was generated to detect DUSP23 (Pacific Immunology) and validated using DUSP23 over-expression and knockdown lysates.

### siRNA reagents

siRNA smartpools for ACP1, ACP2, and DUSP23 (Dharmacon, #019058-00, 008205-00, and J-007909-00), control siRNA smartpool (#001210-02), siRNA to GFP (#P-002048-01) and individual siRNA duplexes toward DUSP23 (du. 01: #J-007909-05 and du.02: #007909-06) were used in experiments at a concentration of 10–20 nM. siRNA knockdown experiments were carried out for 48–72 hours prior to assay or lysis.

### Protein purification and mass spectrometry

Cells from one 15-cm tissue culture dish at 80–90% confluence were lysed in a total volume of 4 ml mammalian cell lysis buffer (MCLB; 50 mM Tris-HCl pH 7.5, 150 mM NaCl, 0.5% Nonidet P40, Roche complete EDTA-free protease inhibitor cocktail) for 20 min at 4 **°**C, and Flag-HA tagged proteins were purified as previously described^14^. Mass spectrometric analysis was carried out as previously described^14^. The spectra were searched using Sequest^43^ against a target-decoy database of human tryptic peptides, and the Sequest identifications were loaded into CompPASS for further processing and analysis^14^,^15^. Raw proteomic data for this publication will be made available upon request.

### CompPASS and bioinformatics analysis to identify interacting proteins for the CCARPs

Mass spectral data were processed using a version of CompPASS analysis that has been modified to assist with identification of high-confidence interacting proteins (HCIPs) that are associated with multiple baits in a network^15^. For analysis of this dataset, Sequest summary files that have been processed based on a 2% false discovery rate were merged for each duplicate run and used to populate a stats table consisting of datasets for each of the dissociators, and datasets for 37 additional unrelated protein complexes from MCF10A cells. Using this stats table, Z-scores and normalized weighted D-scores (WD^N^) were assigned to each of the interacting proteins as previously described^14^,^15^. Interacting proteins with WD^N^ scores > 1.0 (approximately the top 2% WD^N^ scores for all proteins identified by IP/MS) were automatically designated HCIPs. Interactions that had previously been identified in other studies (BioGRID; http://thebiogrid.org/) with Z-scores > 3.48 or with WD^N^ scores > 0.41 (top 5% of WD^N^ scores for all proteins identified by IP/MS) were also included in the finalized lists. Since an interim analysis had determined that proteins with cell-cell adhesion GO terms [list assembled using PROTEOME^TM^ (BIOBASE, http://www.biobase-international.com/product/proteome), AmiGO (The Gene Ontology Consortium, http://amigo.geneontology.org/amigo), and MetaCoreTM (Thompson Reuters, https://portal.genego.com/); Supplemental Table S2] were more likely to interact with multiple baits than were other HCIPs, we reasoned that even with the corrective weighting factor, some valid hits would fall below the stringent top 2% WD^N^ cutoff. To assist with identifying additional relevant hits, we included interacting proteins with cell-cell adhesion GO terms with WD^N^ scores > 0.41 (top 5% of WD^N^ scores for all proteins identified by IP/MS) or Z-score > 3.48. Supplemental Table S1 shows all of the HCIPs and “rescued” interactions, and details which interacting proteins fall into the different “rescued” categories. Supplemental table S4 contains all of the interactions we identified, along with each of the scoring metrics from the CompPASS analysis.

### Immunoprecipitation and western blot analysis

For detection of Tyr phosphorylated adhesion proteins, or to detect changes in the interactions between α- and β-catenin, cells treated with indicated siRNAs or pervanadate (1 mM) as a positive control were lysed in 500 µl MCLB + PhosSTOP (Roche) + pervanadate (1 mM) per two 35 mm wells on ice for 20 min. Clarified lysates were normalized for total protein concentration using BCA assay (Thermo-Fisher Scientific), and 5% of the lysate was reserved for western blot analysis. An antibody directed against the endogenous β-catenin (250 µg) was added together with 30 µl of a 50% prewashed protein A/G bead slurry (Santa Cruz) and rocked for 4–12 hours at 4 **°**C before washing the beads and resuspended the sample in diluted sample buffer (Boston Bioproducts) and boiling in preparation for western blot analysis. Blots of the immunoprecipitated proteins were analyzed for phospho-Tyr signal or for co-precipitating α-catenin using the appropriate antibodies. Blots are representative of three independent experiments, with the exception of the preliminary experiment shown in Fig. 3B, which was carried out once as a prelude to the identification of β-catenin as the candidate protein affected by DUSP23 knockdown.

For validation of IP/MS interactions using western blot analysis, protein complexes were prepared as described above for mass spectrometry and the elutions were prepared by boiling with sample buffer (Boston Bioproducts). Blots were incubated with the appropriate antibody listed above. For IP/MS validation, two independent experiments were run on the same blot next to a control HA-GFP IP.

All western blots were visualized using IRDye 680LT Goat anti-mouse or IRDye 800CW Goat anti-rabbit secondary antibodies (Li-cor #926-68020, 926-32211) and a Li-cor Odyssey imager.

### Membrane fractionation

MCF10A cells were plated at 600,000 cells per 10 cm dish and transfected with 20 nM siRNA for 48 hours. Cells were lysed in 500 µl Subcellular fractionation (SF) buffer (250 mM Sucrose, 20 mM HEPES, pH 7.4, 10 mM KCl, 1.5 mM EDTA, 1 mM EGTA, 1 mM DTT, protease and phosphatase inhibitors) by passing through a 23 Ga needle 10 times using a 1 ml syringe and incubated on ice 20 min. Following centrifugation at 10,000 G for 10 min to remove nuclei, the supernatants containing cytoplasmic and membrane proteins were centrifuged at 100,000 G for 1 hour. The cytoplasmic fraction was removed and added to 100 µl 6X sample buffer for Western blot analysis. Membrane proteins were washed by resuspension in 400 µl SF buffer with a 23 Ga needle as above, and then centrifuged for an additional 45 min at 100,000 G. The pellets containing membrane proteins were resuspended in 100 µl cell lysis buffer (1% Triton X-100, 50 mM HEPES, pH 7.4, 150 mM NaCl, 1.5 mM MgCl_2_, 1 mM EGTA, 100 mM NaF, 10 mM Na pyrophosphate, 1 mM Na_3_VO_4_, 10% glycerol) and added to 20 µl 6X sample buffer for Western blot analysis.

### QPCR analysis of DUSP23 expression

Prior to the generation of the rabbit anti-DUSP23 antibody, DUSP23 knockdown was detected by QPCR using an intron-spanning SyberGreen assay with the primers 5′-CGGCTCCATCGAGACCTAT-3′ and 5′-TTCGTTCGCTGGTAGAACTG-3′ on a Life Technologies 7900HT quantitative PCR instrument.

### Adhesion assays

#### Preparation of Fc-E-cadherin

Fc-E-cadherin was prepared from HEK293 cells that stably express and secrete the fusion protein into the media^44^. Briefly, cells were grown in media containing low IgG serum for 5–10 days prior to harvesting media and purifying Fc-tagged E-cadherin using a protein G column (Pierce). Fractions containing eluted protein were pooled and dialyzed into tris buffered saline, pH 7.4, with 1 mM CaCl_2_, and concentration was determined using a BCA assay (Thermo Scientific).

#### Quantitative adhesion assays

MCF10A cells transduced with siRNAs were briefly trypsinized, quenched with high-serum media, and plated onto glass that was first coated with either 5 µg/ml fibronectin for 30 min, or 50 µg/ml protein A for one hour, followed by 120 µg/ml Fc-E-cadherin for 24 hours. Just prior to plating, 0.5 µg/ml BSA in PBS was incubated with each dish for 30 min to block non-specific binding. After indicated times, non-adherent cells were removed by washing three times with media using a multi-channel pipette. Adherent cells were fixed with 3% PFA and stained with DAPI to visualize nuclei. Large stitched brightfield images (5 × 5 fields) were acquired as above. Images were first filtered using a Gaussian blur with a radius of 2. Filtered images were used to find maxima and plot single points that were then counted as individual cells using ImageJ. The total cells per stitched image were averaged for three wells from experiments carried out on two different days, and plotted using GraphPad Prism (http://www.graphpad.com/scientific-software/prism/).

#### Time lapse imaging

Phase contrast images were acquired at multiple stage positions using a 20X objective lens on a Nikon Ti motorized inverted microscope with Perfect Focus, Nikon linear-encoded motorized stage, and a Hamamatsu ORCA-AG cooled CCD camera controlled by NIS elements image acquisition software. The microscope was fitted with a 37 **°**C Incubation Chamber containing 5% CO_2_.

#### “Hanging drop” cell aggregation assays

MCF10A cells transduced with siRNAs were briefly trypsinized, quenched with high-serum media, and plated into 96-well round bottomed low-attachment dishes (Corning) at a density of 1250 cells in 50 µl. Cells were allowed to form aggregates for 2, 4, and 6 hours before fixation with 4% PFA. Aggregates were transferred to a glass slide using a cut pipette tip and covered with a glass coverslip (Fisher Scientific). Images were taken of the entire slide, up to 15 images per slide, using a 10x objective on a brightfield microscope. Images were analyzed in ImageJ to determine the size of each aggregate. An Otsu threshold was used to generate an outline of each aggregate; each image was inspected for accuracy and outlines were redrawn manually, if necessary. Average sizes of aggregates from 5–8 wells per experimental condition across 2 different days were plotted and analyzed using GraphPad Prism. Error bars represent the 95% confidence interval for the mean.

### Immunofluorescence staining and image analysis

#### Localization of DUSP23

Cells were plated at 100,000 cells per well in 12 well glass-bottomed dishes (Matek) coated with 5 µg/ml fibronectin (Sigma) and allowed to grow for 12–24 hours before fixing with 1:1 methanol:acetone (20 min at −20 **°**C). Cells were blocked/permeabilized using 10% Goat serum (Life Technologies) in IF wash buffer (130 mM NaCl, 7 mM Na_2_HPO_4_, 3.5 mM NaH_2_PO_4_, 7.7 mM NaN_3_, 0.1% BSA, 0.2% Triton X-100, 0.05% Tween 20) prior to incubation with primary antibodies (1:1000 for CTNNB1, 1:250 for all others) for 12–24 hours. Cells were washed in IF wash buffer and incubated in the appropriate goat anti-mouse or goat anti-rabbit secondary antibodies labeled with Alexa Fluor^®^ 488 or 568 (Life Technologies) as indicated. Cells were stained with DAPI (Sigma) to visualize nuclei. Confocal fluorescent Z-series images were acquired using a 20X dry objective on a Nikon A1R Laser Scanning Confocal Microscope equipped with solid state lasers used for excitation with 450/50 and 595/50 filters, or an argon-krypton laser used for excitation with a 525/50 filter. Image acquisition was controlled by Nikon Elements software.

#### Assessment of cell-cell adhesion maturation state

Cells were plated at 100,000 cells per well in 6-well glass bottom plates coated with fibronectin before transfection with a siRNA smartpool to DUSP23 or control siRNAs targeting GFP. After 48 hours, the cells were fixed with 4% paraformaldehyde and stained as described above using an antibody to CTNNB1 with a Goat anti-Mouse secondary antibody conjugated to Alexa Fluor^®^ 488, phalloidin conjugated to Alexa Fluor^®^ 568 to visualize actin structures, and DAPI to visualize nuclei. Large stitched epifluorescence images for assessment of cell-cell adhesion maturation state (7 × 7 fields) were acquired using a 10X objective on a Nikon Ti Automated Inverted Microscope with Perfect Focus Prior Proscan II motorized stage, and a Hamamatsu ORCA-R2 cooled CCD camera controlled by Metamorph software. Through motorized filter wheels and shutters, images were acquired using excitation filters, dichroic, and emission filters from Chroma Technologies. Linescan analysis of β-catenin staining intensity was carried out following an overlaid grid to select cells for junctional assessment to ensure unbiased sampling throughout the well. Linescans were placed manually perpendicular to the cell-cell adhesion through the mid-way point of the adhesion for each junction; analysis was carried out using NIS-Elements AR software (Supplemental Fig. S4A). The staining intensity profile was plotted for each line; average intensity profiles for “mature” and “zipper-like” junctions for both control and DUSP23 knockdown cells are shown in Supplemental Fig. S4B. A junction was scored as “mature” if only one peak of β-catenin staining intensity was present; “zipper-like” junctions had two peaks of β-catenin staining intensity. Over 50 cells were analyzed across two independent experiments; percentages of each type of junction were averaged and plotted using GraphPad Prism.

### Analysis of cell polarization and velocity correlation

Polydimethylsiloxane (PDMS) blocks were used as a barrier to prevent cell seeding on fibronectin coated glass surfaces prior to plating 30,000 H2B-mCherry-MCF10A cells per well as previously described^38^. Cells were transfected with siRNAs to DUSP23 or control siRNAs for 48–72 hours. Prior to imaging, PDMS blocks were removed to reveal the wound region to stimulate migration, and cells were imaged as previously described^38^. Cells were imaged, and automated nuclear tracking and analysis of velocity correlation using timelapse images were carried out as previously described^38^. For polarization assessment, MCF10A-H2B-mCherry cells were incubated with Golgi-GFP (CellLight, Invitrogen) following the manufacturer’s instructions prior to plating on glass coated with 5 µg/ml fibronectin (Sigma) for timelapse imaging as described^38^. Measurement of Golgi orientations were carried out as previously described using images acquired at 0, 4 and 8 h after wounding and were reported in rose plots using MATLAB as described^38^.

## Additional Information

**How to cite this article**: Gallegos, L. L. *et al*. A protein interaction map for cell-cell adhesion regulators identifies DUSP23 as a novel phosphatase for β-catenin. *Sci. Rep*. **6**, 27114; doi: 10.1038/srep27114 (2016).

## Supplementary Material

Supplementary Information

Supplementary Table S1

Supplementary Table S2

Supplementary Table S3

Supplementary Table S4

Supplementary Table S5

Supplementary Movie S1

Supplementary Movie S2

Supplementary Movie S3
