# Supplementary material for: A protein interaction map for cell-cell adhesion regulators identifies DUSP23 as a novel phosphatase for β-catenin
Source: Sci Rep. 2016 Jun 3;6:27114. doi: 10.1038/srep27114 (PMC4891818; doi:10.1038/srep27114)
Supplement: Supplementary Information [file srep27114-s1.pdf]

A protein interaction map for cell-cell adhesion regulators identifies DUSP23 as a novel phosphatase for β-catenin

Lisa Leon Gallegos, Mei Rosa Ng, Mathew E. Sowa, Laura Selfors, Anne White, Ioannis K. Zervantonakis, Pragya Singh, Sabin Dhakal, J. Wade Harper, Joan S. Brugge

Supplemental Figures

Figure S1. Loss of cell-cell adhesion phenotype upon knockdown of CCARP genes from previously published screen data (Simpson, et al., 2008). (A) DIC images from timelapse analysis of scratch wound assay after transfection with control siRNA or siRNA to different CCARP genes. Top images are at 0 min after scratch wound, middle images are at 8 hours post-wounding, bottom images are at 18 hours post-wounding. **(B)** Phalloidin (green) and DAPI (blue) stained wound healing images for additional genes that were not characterized by timelapse analysis.

Figure S2. Validation of selected novel protein interactions between CCAPPs and known cell-cell adhesion proteins. Western blot analysis of anti-HA tag immunoprecipitated protein complexes from MCF10A cells expressing **(A)** HA-GFP, HA-TRIP6, or HA-CSNK1E using anti-TJP2 (top blot) or anti-HA (bottom blot) antibodies; **(B)** HA-GFP, HA-CTNNB1 or HA-CTNNB1 using anti-ARVCF (top blot) or anti-HA (bottom blot) antibodies; **(C)** HA-GFP, HA-CTNNB1 or HA-CTNNB1 using anti-CDH4 (top blot) or anti-HA (bottom blot) antibodies; **(D)** HA-GFP, HA-CTNNB1 or HA-CTNNB1 using anti-PKP4 (top blot) or anti-HA (bottom blot) antibodies; **(E)** HA-GFP, HA-STYXL1, HA-DUSP23, or HA-NEK8 using anti-JUP (top blot) or anti-HA (bottom blot) antibodies; **(F)** HA-GFP, HA-CSNK1G2, or HA-NEK8 using anti-DSG2 (top blot) or anti-HA (bottom blot) antibodies; **(G)** HA-GFP, HA-DUSP23, or HA-NEK8 using anti-CDH3 (top blot) or anti-HA (bottom blot) antibodies; **(H)** HA-GFP or HA-NEK8 using anti-CDH1 (top blot) or anti-HA (bottom blot) antibodies; **(I)** HA-GFP, HA-ACP1, or HA-NEK8 using anti-DSG3 (top blot) or anti-HA (bottom blot) antibodies; **(J)** HA-GFP or HA-ACP1 using anti-CTNNA1 (top blot) or anti-HA (bottom blot) antibodies; **(K)** HA-GFP, HA-CAV1, or HA-RIOK2 using anti-PHB (top blot) or anti-HA (bottom blot) antibodies; **(L)** HA-GFP, HA-ACP1, or HA-DUSP23 using anti-ITGB4 (top blot) or anti-HA (bottom blot) antibodies. Biological duplicate samples are run for each HA-tagged protein.

Figure S3. Analysis of the specificity of the effects of DUSP23 knockdown on the phosphorylation of proteins in the cadherin-catenin complex. (A) Western blot analysis of phospho-tyrosine of δ-catenin (left blots) or α-catenin (right blots) and associated proteins immunoprecipitated from MCF10A cells that were transfected with siRNA to GFP (negative control) or DUSP23, or treated with Vanadate, 1 mM, as a positive control. Upper blots were probed with a generic anti-p-Tyr antibody and the lower blots were probed with antibodies to δ-catenin or α-catenin. * Indicates band at the appropriate molecular weight for α- or δ-catenin; # indicates bands corresponding to IgG. **(B)** Western blot analysis of specific pTyr residues on β-catenin: pTyr 86 (left blots),

pTyr 489 (middle blots), and pTyr 654 (right blots) from whole cell lysates or from immunoprecipitated β-catenin from MCF10A cells transfected with siRNAs to GFP (negative control) or DUSP23. Upper blots were probed with the appropriate anti-pTyr-specific antibody and the lower blots were probed with anti-β-catenin antibody. * Indicates band at the appropriate molecular weight for β-catenin; # indicates bands corresponding to IgG.

Figure S4. Quantification of effects of DUSP23 knockdown on b-catenin membrane fractionation. Quantification of β-catenin protein levels in the membrane fraction (normalized to E-cadherin levels) from immunoblot analysis of membrane and cytoplasmic fractions of cells transfected with siRNA to GFP (control) or DUSP23 (two duplexes; du. 01 and du. 02) corresponding to Fig. 4d. Quantification across three independent experiments showed no significant changes using multiple t-tests.

Figure S5. Cell-matrix adhesion assay and method of assessing mature vs. "zipper-like" cell-cell adhesions. (a) Adhesion assay of MCF10A cells to glass coated with fibronectin. Graph shows quantification of control cells (si-GFP) or DUSP23 knockdown cells (si-DUSP23, two duplexes) remaining adherent to fibronectin after adhesion for 10, 30, and 90 min, as indicated. N = 4 wells across two independent experiments. There are no significant differences amongst the conditions within the same time point; error bars represent S. D. **(b)** Image on the left showing a sub-confluent monolayer of MCF10A cells with both mature and immature cell-cell junctions stained for β-catenin. A line scan through a mature junction (dark blue line) shows one peak of staining intensity (dark blue graph on the right), while a line scan through a "zipper-like" junction (light blue line) shows two peaks of staining intensity (light blue graph on the right). **(c)** Graphs of average β-catenin staining intensity across all junctions measured for control cells and si-DUSP23 cells; black symbols represent mature cell-cell junctions, and grey symbols represent "zipper-like" cell-cell junctions.

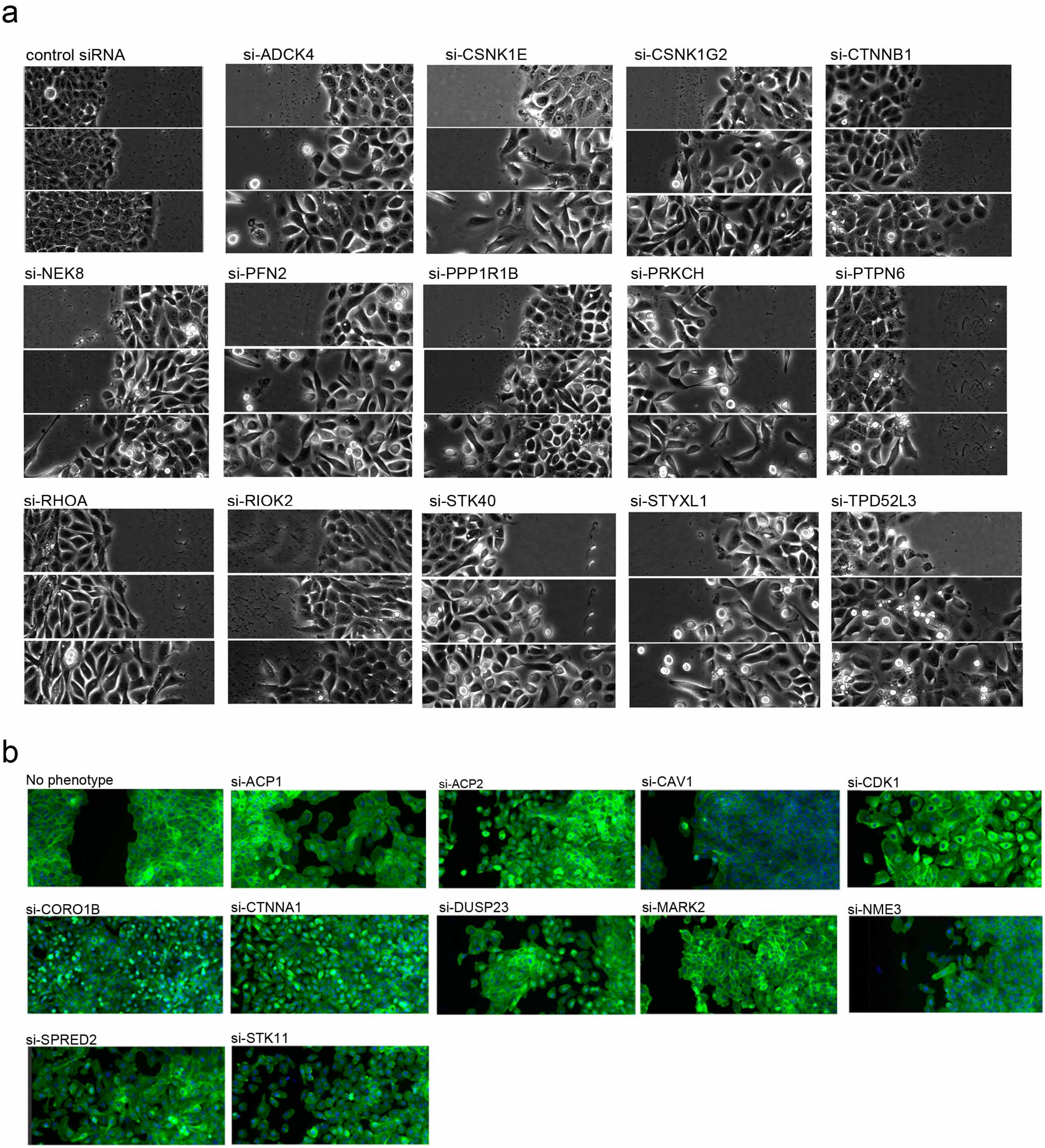
a
control siRNA
si-ADCK4
si-CSNK1E
si-CSNK1G2
si-CTNNB1
si-NEK8
si-PFN2
si-PPP1R1B
si-PRKCH
si-PTPN6
si-RHOA
si-RIOK2
si-STK40
si-STYXL1
si-TPD52L3
b
No phenotype
si-ACP1
si-ACP2
si-CAV1
si-CDK1
si-CORO1B
si-CTNNA1
si-DUSP23
si-MARK2
si-NME3
si-SPRED2
si-STK11

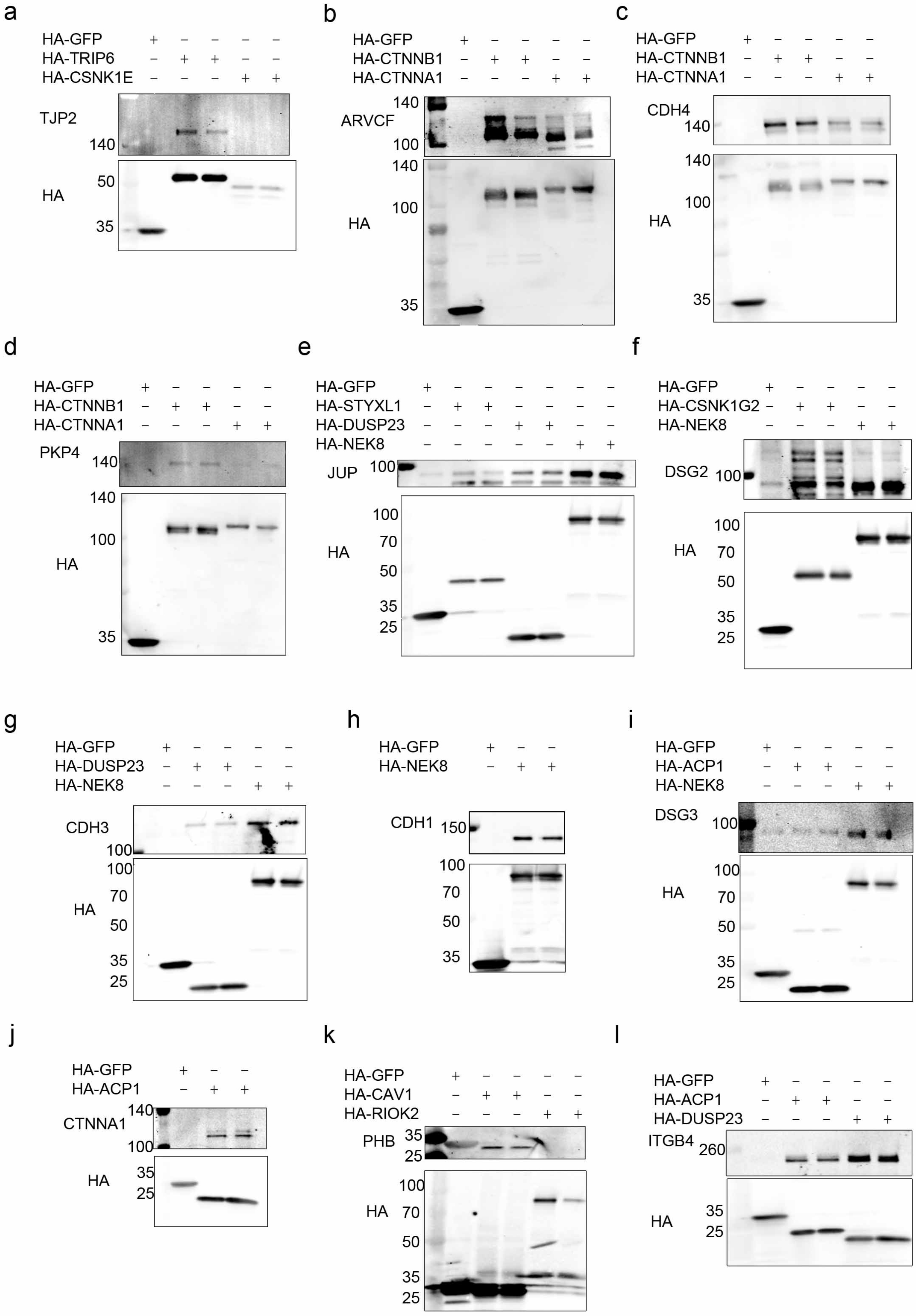
a
HA-GFP + – – – –
HA-TRIP6 – + + – –
HA-CSNK1E – – – + +
TJP2
140
50
HA
35
b
HA-GFP + – – – –
HA-CTNNB1 – + + – –
HA-CTNNA1 – – – + +
ARVCF
140
100
140
100
HA
35
c
HA-GFP + – – – –
HA-CTNNB1 – + + – –
HA-CTNNA1 – – – + +
CDH4
140
140
100
HA
35
d
HA-GFP + – – – –
HA-CTNNB1 – + + – –
HA-CTNNA1 – – – + +
PKP4
140
140
100
HA
35
e
HA-GFP + – – – – – –
HA-STYXL1 – + + – – – –
HA-DUSP23 – – – + + – –
HA-NEK8 – – – – – + +
JUP
100
100
70
50
HA
35
25
f
HA-GFP + – – – –
HA-CSNK1G2 – + + – –
HA-NEK8 – – – + +
DSG2
100
100
70
HA
50
35
25
g
HA-GFP + – – – –
HA-DUSP23 – + + – –
HA-NEK8 – – – + +
CDH3
100
100
70
HA
50
35
25
h
HA-GFP + – –
HA-NEK8 – + +
CDH1
150
100
70
50
35
i
HA-GFP + – – – –
HA-ACP1 – + + – –
HA-NEK8 – – – + +
DSG3
100
100
HA
70
50
35
25
j
HA-GFP + – –
HA-ACP1 – + +
CTNNA1
140
100
HA
35
25
k
HA-GFP + – – – –
HA-CAV1 – + + – –
HA-RIOK2 – – – + +
PHB
35
25
100
HA
70
50
35
25
l
HA-GFP + – – – –
HA-ACP1 – + + – –
HA-DUSP23 – – – + +
ITGB4
260
HA
35
25

a

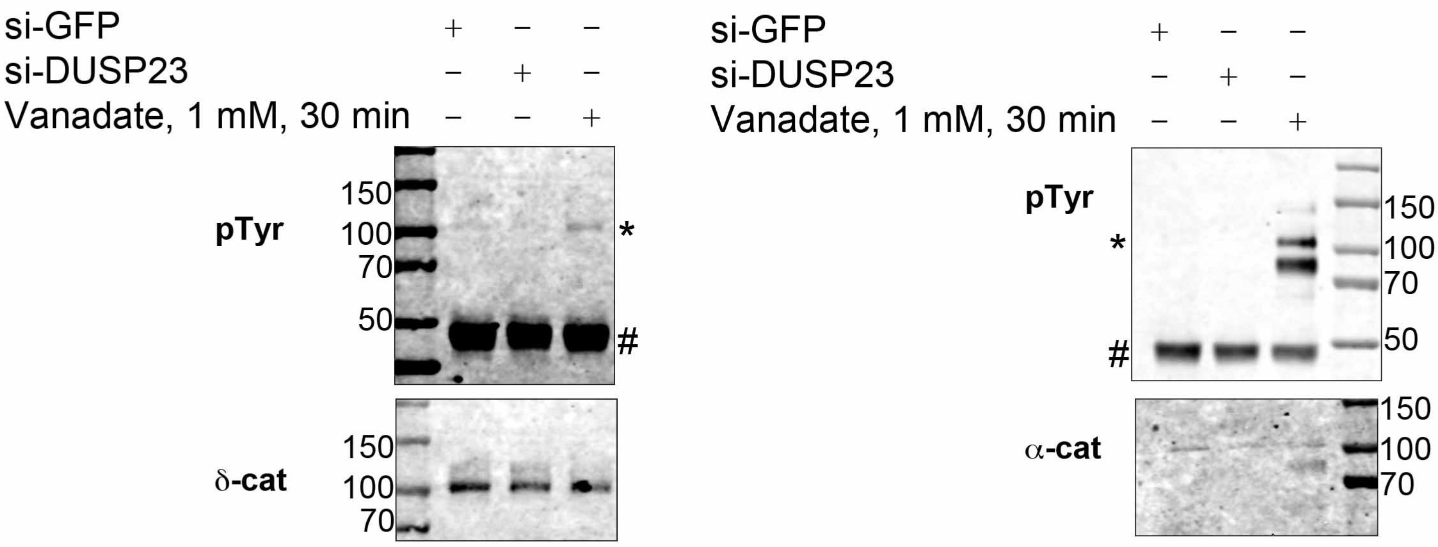
si-GFP
si-DUSP23
Vanadate, 1 mM, 30 min
pTyr
150
100
70
50
*
#
δ-cat
α-cat

b

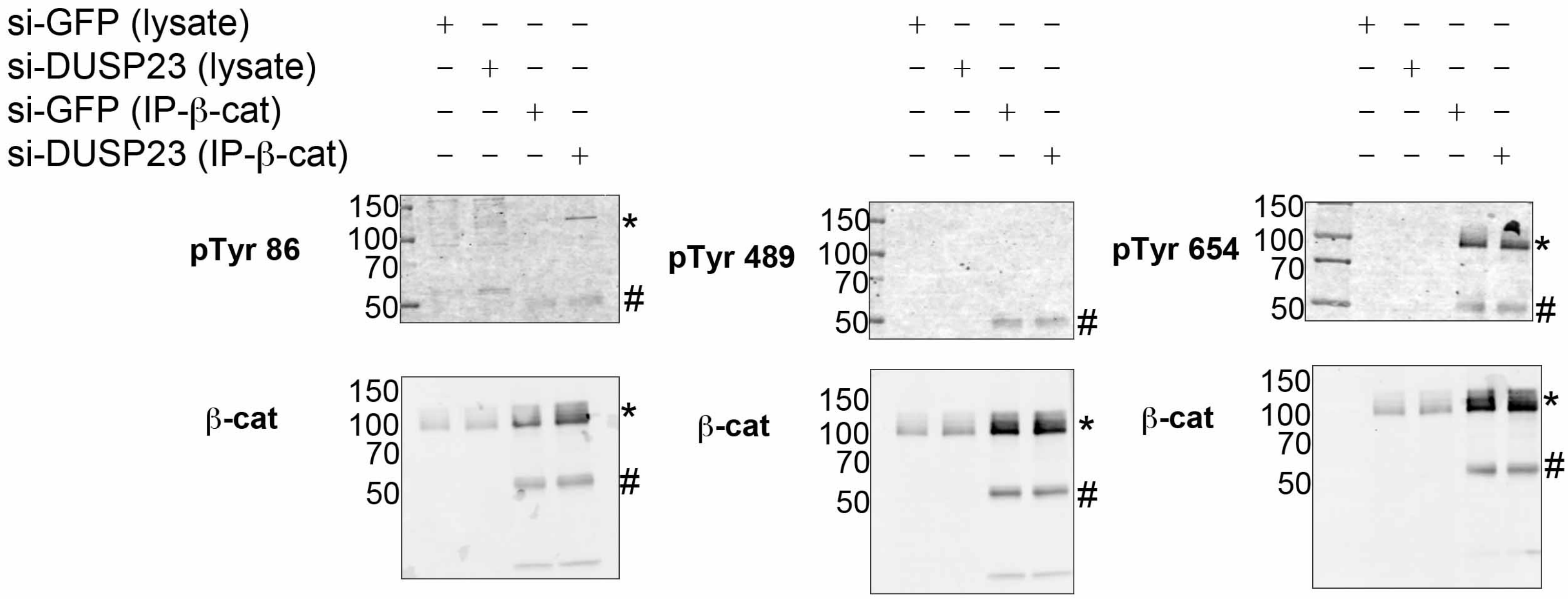
si-GFP (lysate)
si-DUSP23 (lysate)
si-GFP (IP-β-cat)
si-DUSP23 (IP-β-cat)
pTyr 86
pTyr 489
pTyr 654
β-cat
150
100
70
50
*
#

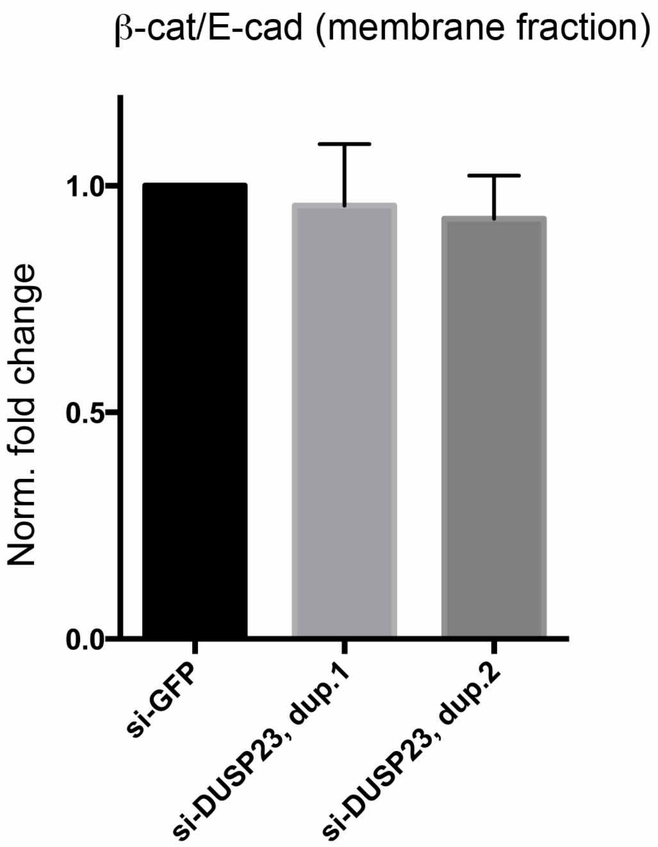
β-cat/E-cad (membrane fraction)
Norm. fold change
1.0
0.5
0.0
si-GFP
si-DUSP23, dup.1
si-DUSP23, dup.2

a

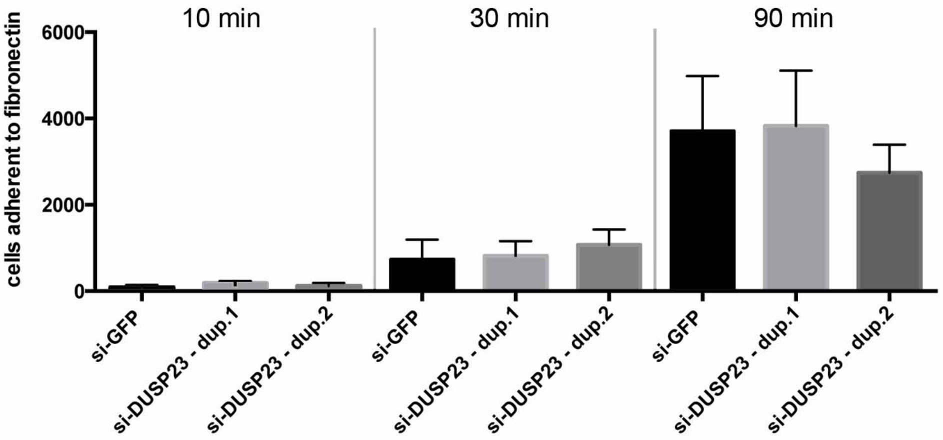
10 min
30 min
90 min
cells adherent to fibronectin
6000
4000
2000
0
si-GFP
si-DUSP23 - dup.1
si-DUSP23 - dup.2

b

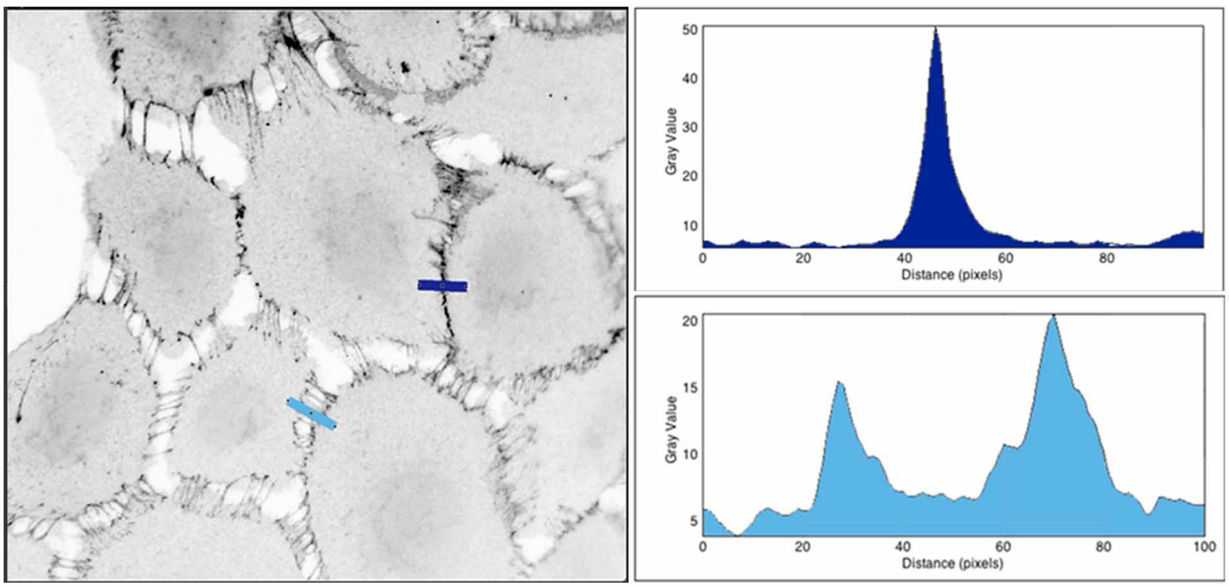
Gray Value
Distance (pixels)
50
40
30
20
10
0
20
40
60
80
15
5
100

c

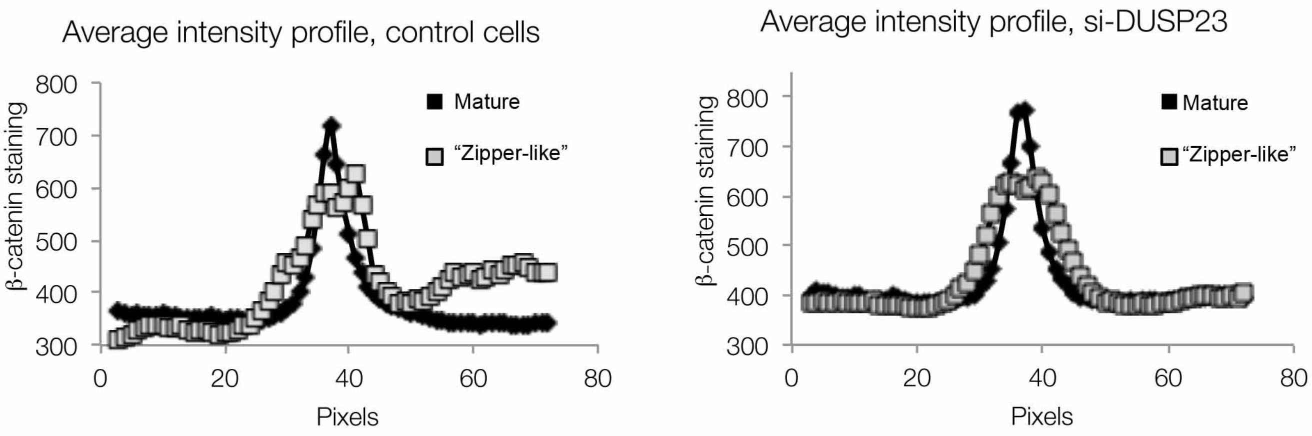
Average intensity profile, control cells
Average intensity profile, si-DUSP23
β-catenin staining
Pixels
Mature
"Zipper-like"
800
700
600
500
400
300
0
20
40
60
80

Dataset Legends

Supplemental Table, S1. Table containing all of the interactions represented in Figure 1. The table also contains CompPASS scores for each interaction.

Supplemental Table, S2. Table containing the list of genes having cell-cell adhesion gene ontology. The databases used are also listed in this table.

Supplemental Table, S3. Comparison of the current study to previous unbiased studies of cell-cell adhesion genes or proteins.

Supplemental Table, S4. Table containing all CompPASS-scored interactors for each bait protein.

Supplemental Table, S5. Table containing all of the expression constructs used in this study.

Supplemental Movie Captions

Supplemental Movie, S1. Movie clip of MCF10A cells transfected with siRNA targeting GFP spreading on glass coated with Fc-E-cadherin. Scale bar indicates 50 μm.

Supplemental Movie, S2. Movie clip of MCF10A cells transfected with siRNA targeting DUSP23, duplex 1, spreading on glass coated with Fc-E-cadherin. Scale bar indicates 50 μm.

Supplemental Movie, S3. Movie clip of MCF10A cells transfected with siRNA targeting DUSP23, duplex 2, spreading on glass coated with Fc-E-cadherin. Scale bar indicates 50 μm.
